# Evaluation of the Ovarian Reserve in Adolescents with Hashimoto’s Thyroiditis Using Serum Anti-Müllerian Hormone Levels

**DOI:** 10.4274/jcrpe.0047

**Published:** 2018-11-29

**Authors:** Ezgi Özalp Akın, Zehra Aycan

**Affiliations:** 1 University of Health Sciences, Dr. Sami Ulus Children’s Health and Disease Training and Research Hospital, Clinic of Pediatrics, Ankara, Turkey; 2 University of Health Sciences, Ankara Dışkapı Yıldırım Beyazıt Training and Research Hospital, Clinic of Pediatric Endocrinology, Ankara, Turkey

**Keywords:** Hashimoto’s thyroiditis, ovarian reserve, anti-Müllerian hormone, adolescents

## Abstract

**Objective:**

This study aims to evaluate ovarian reserve in adolescent girls with Hashimoto’s thyroiditis (HT) by assessment of serum anti-Müllerian hormone (AMH) levels. It was hypothesized that HT decreases ovarian reserve and AMH levels are lower in the HT group.

**Methods:**

Thirty HT patients, aged between 10-18 years, and 30 healthy girls as the control group were enrolled in this cross-sectional study. The mean serum AMH levels of the groups were compared using the Mann-Whitney U test.

**Results:**

There was no statistically significant difference between the patient and the control groups in terms of serum AMH levels. There was a negative correlation between serum AMH and thyroid stimulating hormone (TSH) levels and no correlation between serum AMH and anti-thyroid peroxidase (anti-TPO) or anti-thyroglobulin (anti-Tg) antibody levels.

**Conclusion:**

Our results show that ovarian reserve of adolescent girls, as measured by serum AMH levels, is not affected by HT. Autoimmune damage to the ovaries may take time and the adolescent period may be too early to see these effects. Follow up of the patients for reproductive abnormalities and initiation of prospective studies is recommended.

**What is already known on this topic?**

Hashimoto’s thyroiditis (HT) is the most common disease accompanying premature ovarian failure in adult women. In adolescents, there are only two studies examining ovarian reserve of HT patients. Anti-Müllerian hormone levels of adolescent girls with HT were significantly higher than controls in both studies.

**What this study adds?**

There were no statistically significant differences between the Hashimoto’s thyroiditis (HT) and the control group in serum anti-Müllerian hormone (AMH) concentrations. This study contributes to the limited existing literature on this topic and highlights two important research questions via secondary findings: association of AMH levels and menarche age and determination of AMH levels according to puberty stage.

## Introduction

Hashimoto’s thyroiditis (HT) is an autoimmune disease of the thyroid gland characterized by the lymphocytic infiltration of the thyroid gland and is the most common thyroid disorder in children and adolescents (1). Susceptible individuals who have the combination of abnormalities in cellular immune responsiveness, presence of anti-thyroid auto antibodies, immune susceptibility genes and environmental triggers may develop the disease (1,2).

Anti-Müllerian hormone (AMH) is produced by the granulosa cells of the primary follicles, from fetal life to menopause. Serum AMH levels are correlated with a low antral follicle count (3). Due to its level remaining relatively stable during the menstrual cycle and it not being affected by hormonal feedback mechanisms (3,4), AMH is established as a reliable marker for the quantitative evaluation of ovarian reserve (3,4,5,6). Thyroid hormones are involved in control of the menstrual cycle. Oocytes possess cell surface receptors for triiodothyronine and thyroid hormones affect the actions of follicle-stimulating hormone and luteinizing hormone through steroid biosynthesis. Thyroid dysfunction is associated with menstrual irregularities, anovulation and infertility (7). Premature ovarian failure (POF) describes gonadal failure before the age of 40, defined by clinical and laboratory findings. Abnormalities of cellular immunity and autoimmune processes have a role in the autoimmune etiology of POF. Eighty percent of females with idiopathic POF were reported to have a personal or family history of autoimmune disease, 50% to have high titers of anti-thyroid antibodies and 20% anti-ovary antibodies (8). HT is the most common disease accompanying POF in adult women (8,9,10). Even in women with euthyroid HT, the presence of thyroid autoantibodies is related to female infertility (11,12,13).

In adolescents there are only two studies examining ovarian reserve of HT patients. Results of these two recent studies showed that serum AMH levels of adolescent girls with HT were significantly higher than controls (14,15). In the current study it is hypothesized that HT decreases ovarian reserve and AMH levels are lower in the HT group.

## Methods

Thirty adolescent HT patients aged between 10-18 years were recruited to the study. Thirty euthyroid and autoantibody-negative age-matched adolescents were included in the control group. The patients were diagnosed and followed as HT in Dr. Sami Ulus Children’s Health and Diseases Training and Research Hospital’s Pediatric Endocrinology Outpatient Clinic. Diagnoses of HT were based on clinical evidence, autoantibodies [presence of anti-thyroid peroxidase (anti-TPO) or anti-thyroglobulin (anti-Tg) or both required for the diagnosis], hormone levels and ultrasonography findings. At the time of study all patients had either normal thyroid function or hypothyroidism. Patients with Graves’ disease, hyperthyroidism or irregular menstruation cycles were not included in the study. The control group was composed of adolescent girls who were admitted to our hospital or who had presented to our pediatric outpatient clinic for minor acute illnesses such as upper respiratory tract infections. No patient had a history of chronic disease, chronic drug use or irregular menstruations. An appointment was made for each to assess thyroid function, anti-TPO and anti-Tg autoantibody concentrations.

The study protocol was approved by the Clinical Research Ethics Committee of Zekai Tahir Burak Women’s Health, Training and Research Hospital (with the approval number: 75). Informed consent was obtained from all the subjects and controls prior to enrollment.

All participants were evaluated for pubertal stage, according to Tanner staging (16). Venous blood samples of the patient and control groups were collected for AMH, thyroid stimulating hormone (TSH), free thyroxine (fT4) and anti-TPO and anti-Tg antibody levels. For AMH, blood samples were centrifuged and stored at -20 degrees Celsius and assessed using the AMH Gen II enzyme-linked immunosorbent assay (ELISA; Eastbiopharm, Zhejiang, China) kit. According to the manufacturer, the lowest amount of AMH in a sample that can be detected with a 95% probability is 0.08 ng/mL. The patient group was compared with the control group in terms of serum AMH levels.

The study group was evaluated using thyroid ultrasonography for presence or absence of goiter, thyroid heterogeneity, nodules or any other abnormality by experienced pediatric radiologists. Thyroid volume was measured by the following formula using sonographic measurement of three dimensions in centimeters (a, b, c) of each lobe of the thyroid gland:

Thyroid volume = (a*b*c*0.52) + (a*b*c*0.52)

### Statistical Analysis

All the statistical analyses were conducted using the IBM SPSS for Windows Version 21.0 program (IBM Inc., Chicago, Ill., USA). Results were presented as mean ± standard deviation or median (minimum-maximum). Categorical variables were shown using numbers and percentages. Mann-Whitney U test was performed to confirm the difference between the two groups; Kruskal-Wallis test was used for more than two groups as non-parametric tests. A chi-square test was used to confirm the relationship of the categorical variables. Spearman’s correlation coefficient was used to determine the relationship between the numeric variables. P values <0.05 were considered statistically significant.

## Results

The patient and the control groups had the same mean [± standard deviation (SD)] age which was 14.4 (±1.85) years. The mean (±SD) follow up time for the patient group was 8.5 (±4.5) months. The minimum Tanner stage of puberty was 2 and median stage was 5 in both groups. There were no significant differences between the groups in terms of pubertal stage and age. Ten of the 60 subjects (five from the patient group, five from the control group) had not reached menarche at the time of study. Excluding these 10 subjects, the mean menarche age of the patient group was significantly earlier than the control group, (11.4±0.86 versus 12.4±1.04, p=0.001).

Median serum AMH level of the patient group was 1.7 ng/mL (minimum 0.5 ng/mL, maximum 5.1 ng/mL), lower than in the control group which was 1.8 ng/mL (minimum 0.29 ng/mL, maximum 5.5 ng/mL). However this was not statistically significant (p=0.784).

There was no statistically significant difference between groups in terms of TSH and fT4 levels, probably because 86% of the patient group was on levothyroxine treatment. Four newly diagnosed cases had subclinical hypothyroidism. Twenty-six cases were euthyroid. There was no subject with overt hypothyroidism. There was a significant negative correlation between serum AMH and TSH levels in the total sample: r=-0.29, p=0.02 and in the study group: r=-0.29, p=0.02. When the control group alone was assessed this relationship was very close to significance: r=-0.36, p=0.05. There was no correlation between serum AMH and anti-TPO or anti-Tg levels.

Forty-three of 60 subjects (71%) were at stage 5 puberty and their median serum AMH level was higher than the median AMH levels of girls at puberty stages 3 and 4 (Table 1), but the difference was not statistically significant.

Forty percent of the patient group had goiter, 93.3% had thyroid tissue heterogeneity and 43.3% had septation on the thyroid ultrasonography imaging. Goiter, heterogeneity or septation of the thyroid gland were not found to be associated with serum AMH levels.

## Discussion

This study showed that no statistically significant differences were present in terms of serum AMH levels between the HT adolescents and the control group. Serum AMH levels were negatively correlated with serum TSH levels for patients and for the subject plus control groups.

An unexpected result reported in a recent study on adults, conducted to evaluate the ovarian reserve of 32 women with HT as compared to 49 healthy females was that serum AMH levels were higher in women with HT (17). The authors suggested that this finding may be due to polycystic ovary syndrome, which may share a common etiologic linkage with autoimmunity and HT. Another aspect of this study was the lack of a statistically significant difference between the study and control patients in terms of antral follicle count.

Only two studies on ovarian reserve of HT adolescents have been reported to date. Results of these two recent studies showed that serum AMH levels of adolescent girls with HT were significantly higher than controls. In one of these studies (14), 30 newly diagnosed HT adolescents were enrolled as the study group and compared to healthy adolescents. In both our study and in Pirgon et al’s (14) study the sample had no menstural irregularities. Our study is different in terms of follow up time of the patient group (mean 8.5±4.5 months), from Pirgon et al’s (14) study in which the cases were just diagnosed. So there was a longer time for the autoimmune process to affect the ovaries. Higher serum AMH levels and lower serum anti-oxidant levels in euthyroid HT subjects were reported in the second study on this topic (15). The discrepancy of serum AMH levels between our study and these former reports may be due to differences in the thyroid status of the HT groups and the duration of autoimmune thyroiditis. However all three studies support the finding that ovarian reserve of HT patients is not decreased in adolescence when assessed by serum AMH concentrations.

Serum AMH concentration varies during a woman’s lifetime. Hormone expression from the ovaries starts with fetal life, reaching the maximum level at puberty, starts to decrease in adulthood and disappears following the menopause. This hormone is expressed by the granulosa cells of the primary follicles, specifically in the preantral and small antral follicles. The expression is decreased in the large antral follicles (18,19). In our study the difference between the serum AMH concentrations at different pubertal stages were not statistically significant but the higher AMH levels at puberty stage 5 and lower levels in earlier stages were noteworthy findings. The number of cases in our study group at puberty stages 2 (n=5), 3 (n=4) and 4 (n=8) was small whereas 43 of the 60 cases were at stage 5 puberty. A larger group of subjects in earlier stages of puberty are needed to examine the relationship between puberty stages and AMH concentration.

The variation of serum AMH concentrations during puberty have been evaluated in a previous study (20). In this well-planned study, serum AMH levels of 381 girls, aged eight years, were recorded. Thirty-nine of these girls had telarche and their serum AMH concentrations were significantly lower than those who had not attained thelarche (p=0.001). In the longitudinal part of this study, 32 girls were followed and their AMH concentration was recorded at seven, nine and 11 years of age. Between seven and nine years, the concentration of serum AMH was increased. This was explained by the transition of AMH-silent primordial follicles into AMH-secreting, small antral follicles. Between the ages of nine and 11 years, serum AMH concentration decreased, which was explained by the transition of the small antral follicles into large antral follicles which secrete less AMH than small antral follicles. These findings support the concept that puberty is a special stage of life which may require AMH reference ranges to be set for puberty stage rather than for chronological age.

Serum AMH concentrations were negatively correlated with serum TSH concentrations, but not correlated with anti-TPO and anti-Tg autoantibodies in the present study. Independent of autoimmunity, subclinical hypothyroidism may affect both ovarian function and reserve. Tuten et al (17) reported that serum AMH and both anti-TPO and anti-Tg autoantibody concentrations were positively correlated, while serum AMH and TSH levels were not in adult HT patients. In a large cross sectional study from Belgium, women were divided into low, middle and high ovarian reserve categories according to serum AMH levels (21). There was no significant difference in the prevalence of positive anti-TPO antibodies between the different ovarian reserve categories, a finding compatible with the present study.

In our study, mean menarche age of the patient group was significantly lower than the control group. It is possible that early menarche may be related to early menopause or POF. The relationship between autoimmunity and early menarche is unclear. In a prospective study, 46 children and adolescents with HT were followed for six years and the mean age of menarche was not found to be different from normal children (22). Another autoimmune disorder, celiac disease, is associated with late age of menarche and this has been attributed to autoimmunity and micro- and macro-nutrient deficiencies (23). In a retrospective study, anti-nuclear antibody prevalence in postmenopausal women was found to be associated with late menarche age (24). Early menarche is associated with cardiovascular risk factors, obesity and breast cancer. Therefore these patients should be followed for these entities.

Our study is one of the firsts conducted on AMH levels of children with HT. Including our report, currently there are only three studies investigating AMH concentrations of adolescent with HT, and none of them showed ovarian reserve impairment. However, follow up time and thyroid status differ between these studies. More comprehensive, prospective studies with higher sample sizes are required to investigate the relationship between HT and ovarian reserve in adolescents. Our study expands the limited existing literature on ovarian reserve in HT adolescents and highlights two other important research areas. These are the association of AMH concentrations and menarche age and AMH concentrations according to pubertal stage.

### Study Limitations

The major limitations of our research is the small sample size and the cross-sectional design. Long-term follow up is needed to see if there will be any ovarian reserve impairments due to autoimmunity in time.

## Conclusion

In conclusion, our study showed that the ovarian reserve of adolescents, evaluated by serum AMH concentration, was not affected by HT. It is possible that autoimmune damage to the ovaries takes time and adolescence may be too early in the process to see the effects. Follow up of adolescents with HT for reproductive abnormalities and prospective studies starting from childhood will be enlightening.

## Figures and Tables

**Table 1:**
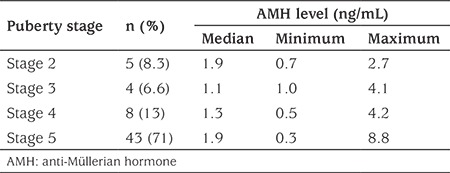
Serum anti-Müllerian hormone levels of the sample according to Tanner puberty stages
